# Quantifying spatial accessibility in public health practice and research: an application to on-premise alcohol outlets, United States, 2013

**DOI:** 10.1186/s12942-018-0143-y

**Published:** 2018-06-27

**Authors:** Hua Lu, Xingyou Zhang, James B. Holt, Dafna Kanny, Janet B. Croft

**Affiliations:** 1 Division of Population Health, National Center for Chronic Disease Prevention and Health Promotion, Centers for Disease Control and Prevention (CDC), 4770 Buford Highway, N.E. Mailstop F-78, Atlanta, GA 30341 USA; 2 Economic Research Service, U.S. Department of Agriculture, Washington, DC USA

**Keywords:** Network analysis, Spatial accessibility, Alcohol outlet

## Abstract

**Objective:**

To assess spatial accessibility measures to on-premise alcohol outlets at census block, census tract, county, and state levels for the United States.

**Methods:**

Using network analysis in a geographic information system, we computed distance-based measures (Euclidean distance, driving distance, and driving time) to on-premise alcohol outlets for the entire U.S. at the census block level. We then calculated spatial access-based measures, specifically a population-weighted spatial accessibility index and population-weighted distances (Euclidean distance, driving distance, and driving time) to alcohol outlets at the census tract, county, and state levels. A multilevel model-based sensitivity analysis was conducted to evaluate the associations between different on-premise alcohol outlet accessibility measures and excessive drinking outcomes.

**Results:**

The national average population-weighted driving time to the nearest 7 on-premise alcohol outlets was 5.89 min, and the average population-weighted driving distance was 2.63 miles. At the state level, population-weighted driving times ranged from 1.67 min (DC) to 15.29 min (Arizona). Population-weighted driving distances ranged from 0.67 miles (DC) to 7.91 miles (Arkansas). At the county level, population-weighted driving times and distances exhibited significant geographic variations, and averages for both measures increased by the degree of county rurality. The population-weighted spatial accessibility indexes were highly correlated to respective population-weighted distance measures. Sensitivity analysis demonstrated that population weighted accessibility measures were more sensitive to excessive drinking outcomes than were population weighted distance measures.

**Conclusions:**

These results can be used to assess the relationship between geographic access to on-premise alcohol outlets and health outcomes. This study demonstrates a flexible and robust method that can be applied or modified to quantify spatial accessibility to public resources such as healthy food stores, medical care providers, and parks and greenspaces, as well as, quantify spatial exposure to local adverse environments such as tobacco stores and fast food restaurants.

**Electronic supplementary material:**

The online version of this article (10.1186/s12942-018-0143-y) contains supplementary material, which is available to authorized users.

## Background

Quantifying spatial accessibility in public health practice is essential for evaluating population exposure to local environments (e.g., alcohol and tobacco outlets or public parks) and population access to health care resources (e.g., primary care clinics or trauma hospitals). Three approaches have been reviewed by Zhang et al. [1] and summarized for application to the measurement of alcohol outlet density by CDC [2]. Table 1 summarizes the common metrics for quantifying spatial accessibility and basic relationships among them. Two commonly used approaches for quantifying spatial accessibility are distance-based and container-based. The spatial interaction (or gravity) model-based spatial accessibility index uses both distance-based metrics and the container concept. Population-weighted accessibility metrics are based on the spatial interaction model-based spatial accessibility index, but at the same time account for the uneven population distribution within a study area. Population-weighted distance metrics use distance-based metrics and at the same time use the spatial interaction model-based spatial accessibility index to construct differential probability access to destinations and further account for the uneven population distribution within a study area. The most complex population-weighted distance metrics aim to borrow the strengths and at the same time minimize the limits of classic distance-based and container-based metrics, and integrate the power and flexibility of the spatial interaction model-based approach and account for uneven population distributions.

**Table 1:** Metrics for quantifying spatial accessibility

*Distance-based metrics:* Distance to nearest one destination or a group of nearby destinations - Euclidian distance (also known as flight or straight-line) - driving distance (also known as street network distance, which accounts for street network lengths and connectivity) - driving time (which further accounts for speed limits for each segment of the street network)	*Container-based metrics:* Number of destinations within a pre-specified area or spanning distance or some spatial density measures including but not limited to: - per 1000 population - per area unit (e.g. square miles) - per road miles
*Spatial interaction (gravity) model-based metrics:* - Choose distance metrics - Define distance decay function and distance decay parameter - Specify the destination choice set within an area or spanning distance - Construct spatial accessibility index	
*Population-weighted accessibility metrics:* Aggregate spatial accessibility index weighted by population - Euclidian distance - driving distance - driving time	*Population-weighted distance metrics:* Use spatial accessibility index to define differential probability access to destinations in the choice set and further weighted by population - Euclidian distance - driving distance - driving time

Distance-based measures, such as distance to nearest outlets, are intuitive and relatively easy to generate, but often lack the ability to quantify and incorporate the potential geographic clustering effects of spatial destinations. The three most common distance-based metrics are listed in Table 1.

The container-based approach is most commonly used to assess outlet density for a predefined area (container). Some common container-based metrics are the number of outlets per square mile or per road mile, or within a predefined spanning distance (Table 1), but often there is an underlying assumption of equal accessibility within this predefined area. There could also be substantial boundary or edge effects, such that outlets near the predefined area boundaries could be either included or excluded in the density calculation. The spatial interaction model–based approach assumes the spatial association or interaction between spatial or geographic entities are proportional to their mass sizes and inversely proportional to their distances. It takes four basics steps to construct a spatial accessibility index, as detailed in Table 1.

The spatial interaction model-based spatial accessibility index is often used by geographers because of its flexibility and robustness in quantifying spatial accessibility. However, interpretations of these indices are not intuitive for public health practitioners and communities. Population-weighted accessibility (PWA) metrics have similar interpretation challenges in public health practice.

The population-weighted distance (PWD) metrics, a form of spatial accessibility measure developed by Zhang et al. [1] accounts for spatial clustering of outlets, overcomes the unrealistic equal access assumption and potential edge effects of the container-based approach, and also uses an intuitive form of a distance-based measure. In addition, PWD also accounts for uneven population distribution for the geographic area of interest. The flexibility of PWD measures makes them applicable to individual persons or households, as well as for a geographic area. The individual-based PWD measures could be applied in specific public health analyses with individual records. The area-level PWD measures are based on the smallest census geographic units (census blocks); thus, PWD measures could be conveniently aggregated to any larger geographic levels as needed while avoiding the modifiable unit area problem [3]. Despite these potential advantages, PWD measures may be sensitive to the choice of the original distance measures upon which the resulting population-weighted measures are computed. The spatial interaction model to calculate population-weighted accessibility and accessibility index is described in Zhang et al. [1].

In the United States, the census block is the basic unit of census geographic hierarchy (https://www.census.gov/geo/reference/hierarchy.html). We used census block-level population as our demand area population. By using the population at finest level of census geography that is available in the United States, we minimized the geographic aggregation error for population data described as Source A type of error in Hillsman and Rhoda [4]. Spatial access metrics based on higher levels of census geography than census blocks (e.g. census tract, ZIP Code, or county), even by using population weighted centroids to estimate demand population locations, could still introduce substantial spatial aggregation errors or Source A type errors. In addition, the census block-based spatial access metrics provide the flexibility to aggregate the metrics to any high-level geography that could be linked to geocoded individual or aggregated health outcomes. For example, we could easily aggregate census block-based spatial access metrics to higher-level census geographic units, such as census tract, county, ZIP Code, school district, congressional district, or other administrative or customized geographic units, such as hospital service areas, hospital referral regions, and primary care service area. However, there were no studies that quantify spatial accessibility based on different census block-level distance measures (Euclidian distance, driving distance and driving time) and evaluate their correlations between population-weighted spatial accessibility index and population-weighted distance metrics.

In this study, we selected on-premise alcohol outlets because of significant public health interest in excessive alcohol use. Greater alcohol outlet density is associated with increased alcohol consumption and related harms [5]. Excessive alcohol consumption is responsible for 88,000 deaths annually in the United States [6] and accounted for $249 billion in economic costs in 2010 [7].

An alcohol outlet is defined as a place where alcohol may be legally sold to a buyer to consume there (e.g., on-premise outlets, such as bars or restaurants) or elsewhere (e.g., off-premise outlets, such as liquor stores) [5]. The on-premise alcohol outlet data that we obtained from HSIP GOLD is a point dataset that has been geocoded to the street address of the outlets. The point-level data eliminated a location error when geocoding data to ZIP Code area centroid. Regulating alcohol outlet density, which is typically defined as the number of alcohol outlets in a given area, is an effective strategy for the prevention of excessive alcohol consumption and related harms [8]. The three general approaches for quantifying spatial accessibility described previously can be applied to the measurement of alcohol outlets [2]. In the container-based approach, the number of alcohol outlets in a given area is calculated. Different denominators can be used as follows: by population size (number of outlets per 1000 population); by area size (number of outlets per square mile); or by road mile length (number of outlets per road mile). Commonly, measurements of alcohol outlet density have used container-based approaches, in which the number of outlets is divided by the population size of a particular area or by the land area itself [9]. However, these approaches could suffer from boundary and edge effects, especially for small geographic areas, and do not directly consider the spatial accessibility between alcohol outlets and the population [2]. It can result in overestimates for small areas with large numbers of alcohol outlets, or underestimates for small areas with small numbers or no alcohol outlets.

The distance-based approach calculates the distance to the nearest specified number of alcohol outlets. The distance can be calculated based on Euclidean distance, driving distance through a street network, and driving time through a street network with speed limits. The advantage to this method is that it is intuitive for point-to-point units; however, it is less commonly used in alcohol outlet density studies. The limitation for the distance-based approach is that it ignores the possibility of multiple potential alcohol outlet destinations within an area. An alternative approach is to average the distances to a set of nearest (e.g. five or seven) alcohol outlets. However, this approach suffers from ignoring the unequal probability of access to any outlet. The population-weighted distance approach uses population as a weight to account for uneven population distributions. It incorporates unequal probability of access to nearby alcohol outlets, defines the choice set of alcohol outlets guided by human behavioral theory, and is able to account for alcohol outlet size (if data are available) and spatial clustering [9].

The objectives of this study are to use a comprehensive dataset of locations of on-premise alcohol outlets in the United States to create a population-weighted accessibility index and population-weighted distance metrics to quantify population access to on-premise alcohol outlets at the census block, census tract, county, and state levels for the entire U.S. Specifically, we use three different distance metrics: Euclidian (flight) distance, driving distance and driving time, to construct a spatial accessibility index to generate three population-weighted accessibility metrics and three more intuitive population-weighted distance metrics. We then compared and evaluated their similarities and correlations. This spatial accessibility modeling framework could be conveniently applied to any other countries or places with hierarchical census geography, such as, census output areas, super output areas, electoral wards, and electoral divisions in the UK; villages, street districts, towns, counties, cities, and provinces in China.

## Methods

### Data sources

1. On-premise alcohol outlets

We obtained geocoded data for on-premise alcohol outlets from the Homeland Security Infrastructure Program (HSIP) GOLD 2013 dataset [10]. It included 210,482 drinking establishments (HSIP terminology to represent on-premise alcohol outlets) in the 50 States and DC, with state averages ranging from 435 (DE) to 16,942 (TX) drinking establishments. The North American Industry Classification System (NAICS) is the standard used by Federal statistical agencies in classifying business establishments for the purpose of collecting, analyzing, and publishing statistical data related to the U.S. business economy. We used NAICS code 7224 and all subcategory NAICS codes of 7224 for Drinking Places (Alcoholic Beverages). The types of on-premise alcohol outlets in these NAICS codes include bars and lounges, drinking places, nightclubs, eating places, restaurants, hotels and motels, bowling centers, and recreation clubs. This dataset provided the exact street addresses of on-premise alcohol outlets.

2. Geographic unit of analysis and population demographics

We used 2010 census blocks as our geographic unit of analysis. Census blocks are the smallest unit in the census geographic hierarchy, thus census block-level measures could be aggregated to any geographic levels of interest in a very flexible way and minimize the effects of the modifiable area unit problem. We included 6,207,027 census blocks with 2010 populations greater than zero in this study. The geometric centroid of the census block was used to calculate the distance to the location of on-premise alcohol outlets. The 2010 census population count was used as the weight to calculate the population-weighted spatial accessibility index, population-weighted driving distance, population-weighted driving time and population-weighted Euclidean distance at each geographic level.

3. Street network data

We used Esri Data and Maps 10.2 U.S. and Canada Detailed Streets network dataset (Esri, Redlands, CA). These data provide distance and travel times for each street segment that was used in our network analyses.

### Measures

1. Distance metrics

We used ArcGIS 10.2.1 Network Analyst (Esri, Redlands, CA) to calculate the nearest 7 on-premise alcohol outlets for each populated census block. This tool produced a driving distance and a driving time for each origin (census block) and destination (on-premise alcohol outlet) pair. There were 2118 census blocks that did not have access to a street network, and thus were excluded in the driving distance and time calculations. We also calculated the Euclidian distance for each census block to its 7 nearest on-premise alcohol outlets using all census blocks in the U.S. We used SAS GEODIST function to calculate the Euclidian distance between census block centroids and alcohol outlets (SAS, Cary, North Carolina).

2. Population-weighted spatial accessibility index

We used the spatial interaction model to calculate the population-weighted spatial accessibility index as described in the introduction. Because the size of each on-premise alcohol outlet is unknown, we used a weight of 1 to treat each outlet equally. The Census block-level spatial accessibility index was calculated as:

$$A_{i} = \mathop \sum \limits_{j = 1}^{7} \frac{1}{{d_{ij} }}$$

where *i* is *i*th census block, *j* is *j*th on-premise alcohol outlet and n = 7 was used for 7 nearest outlets in our study. The population-weighted spatial accessibility index for geographic units larger than census blocks was calculated as:

$$PWA_{k} = \frac{{\mathop \sum \nolimits_{i = 1}^{N} Pop_{i} *A_{i} }}{{Pop_{K} }}$$

where *N* is the number of census blocks within a geographic area *k* (e.g. county), Pop_*i*_ is the *i*th census block’s total population, and Pop_*k*_ is the total population for the geographic area *k*.

3. Population-weighted driving distance/time, Euclidian distance

We used distances and times from the distance-based measures along with block-level populations to calculate the population-weighted driving distance and population-weighted driving time for each census block. Because of the massive size of the data involved in the study, we stratified the U.S. census blocks into 17 smaller regions for computational purposes to perform the network analysis and then merged the results into one data file.

Population-weighted driving distance/time for census block *i* to 7 nearest on-premise alcohol outlets is calculated as:

$$PWD_{i} = {{\mathop \sum \limits_{j = 1\sim7} \left( {Pop_{i} *P_{ij} *d_{ij} } \right)} \mathord{\left/ {\vphantom {{\mathop \sum \limits_{j = 1\sim7} \left( {Pop_{i} *P_{ij} *d_{ij} } \right)} {Pop_{i} }}} \right. \kern-0pt} {Pop_{i} }} = \mathop \sum \limits_{j = 1\sim7} \left( {P_{ij} *d_{ij} } \right)$$

where P_ij_ is the probability that a resident at a census block i will choose to visit an alcohol outlet j:

$$P_{ij} = {{\frac{1}{{d_{ij}^{1} }}} \mathord{\left/ {\vphantom {{\frac{1}{{d_{ij}^{1} }}} {\mathop \sum \limits_{j = 1}^{7} \frac{1}{{d_{ij}^{1} }}}}} \right. \kern-0pt} {\mathop \sum \limits_{j = 1}^{7} \frac{1}{{d_{ij}^{1} }}}} = A_{ij} /A_{i}$$

The probability was derived from Huff Model 1963 [11].

Population-weighted driving distance/time for area *k* is calculated as:

$$PWD_{k} = \frac{{\mathop \sum \nolimits_{i = 1}^{N} Pop_{i} *PWD_{i} }}{{Pop_{K} }}$$

Using these methods, we calculated the population-weighted accessibility (PWA) index and population-weighted distance (PWD) distances (by driving distance, driving time, and Euclidian distance) to the 7 nearest on-premise alcohol outlets at the census block level. Then, we calculated the same spatial accessibility measures at U.S.-, state-, county- and census tract-levels respectively.

### Data analysis

We compared the 6 spatial accessibility measures using Pearson correlation coefficients at the census block level. Specific measures that we analyzed—all at the county level—were driving distance, driving time, and Euclidean distance to the one nearest on-premise alcohol outlet; driving distance, driving time, and Euclidean distance to the nearest 7 on-premise alcohol outlets in A_ij_ = 1/(d_ij_^^β^) with a distance decay parameter β = 1.

We used county-level spatial accessibility results linked with the 2013 National Center for Health Statistics (NCHS) urban–rural classification scheme for counties [12] and calculated spatial accessibility by urban and rural counties to determine whether urban/rural status was associated with spatial accessibility measures. The NCHS urban–rural classification for counties classified U.S. counties into the following 6 categories: (1) large central metro, defined as counties in a metropolitan statistical area (MSA) of at least 1 million residents that contain the entire population of the largest principal city of the MSA, or are completely contained within the largest principal city of the MSA, or contain at least 250,000 residents of any principal city in the MSA; (2) large fringe metro, defined as counties in an MSA of 1 million or more residents that does not qualify as a large central metro; (3) medium metro, defined as counties in an MSA of 250,000–999,999 residents; (4) small metro, defined as counties in an MSA of less than 250,000 residents; (5) micropolitan, defined as counties in a micropolitan statistical area; and (6) noncore (often called rural), defined as counties not in MSAs or micropolitan statistical areas. Categories 1–4 are metropolitan counties and categories 5–6 are nonmetropolitan counties.

### Sensitivity analysis

We conducted a sensitivity analysis via linking population weighted spatial accessibility metrics to drinking outcomes from a nationwide health survey. We first calculated the county level population weighted spatial accessibility measures based on driving distance, driving time and Euclidian distance to the nearest 7 on-premise alcohol outlets. We then linked these county level alcohol outlet accessibility measures to individual binge drinking (4 or more drinks on an occasion for women, 5 or more drinks on an occasion for men) and heavy drinking outcomes (8 or more drinks per week for women, 15 or more drinks per week for men) from the restricted 2013 Behavioral Risk Factor Surveillance System (BRFSS) data with survey respondents’ county residence identifiers. With this linked dataset, we constructed a series of multilevel logistic models for both drinking outcomes and estimated the odd ratios associated with alcohol outlet access measures. These multilevel logistic models also included individual age, gender, race/ethnicity, education and income; county-level poverty; and county- and state-level contextual effects (specified by county-level and state-level random effects). The adjusted ORs were used to detect the significant associations between alcohol outlet access and drinking outcomes and to measure their sensitivity to drinking outcomes. A OR larger than one means an increased risk for drinking behaviors, and a larger OR means more sensitive to drinking outcomes. SAS Proc GLIMMIX was used to implement these multilevel logistic models.

## Results

In 2013, the average driving time to the nearest on-premise alcohol outlets (of the nearest 7 choices) for the overall U.S. population was 5.89 min and the average driving distance was 2.63 miles. For all census blocks, the median population-weighted driving time was 4.72 min; population-weighted driving distance was 1.99 miles; and population-weighted Euclidian distance was 1.36 miles (Table 2).

**Table 2:** Census block-level population-weighted spatial accessibility measures to 7 nearest on-premise alcohol outlets, United States, 2013

Measures	Number of census blocks	Mean	Standard deviation	Minimum	Median	Maximum
*Population*-*weighted accessibility*						
Driving time	6,204,909	2.46	3.09	0.00	1.48	48.98
Driving distance	6,204,909	6.14	8.52	0.00	3.51	500.02
Euclidian distance	6,207,027	8.81	11.77	0.00	5.16	439.78
*Population*-*weighted distance*						
Driving time (min)	6,204,909	9.29	11.56	0.14	4.72	1321.00
Driving distance (miles)	6,204,909	4.41	6.00	0.01	1.99	531.27
Euclidian distance (miles)	6,207,027	3.25	5.39	0.02	1.36	457.59

**Fig. 1:**
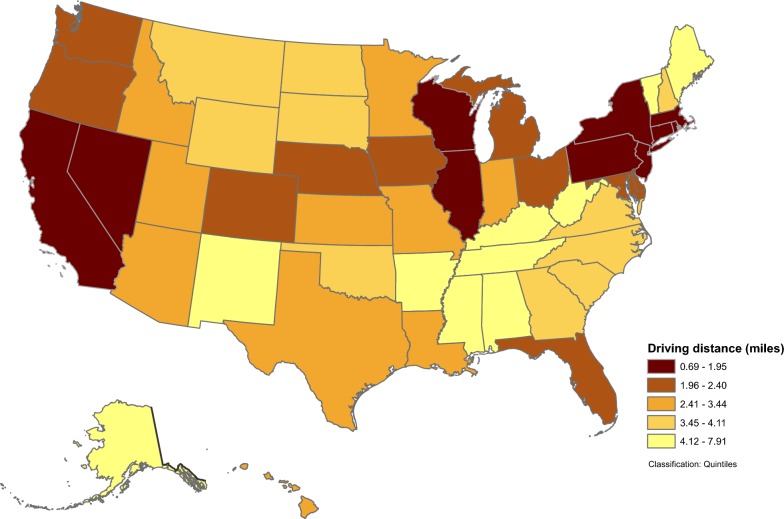
Population-weighted driving distance to 7 nearest on-premise alcohol outlets, by states, United States, 2013

**Fig. 2:**
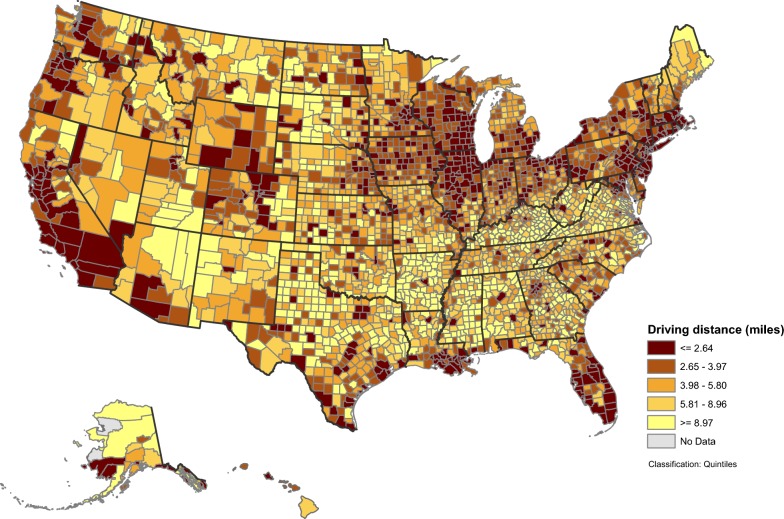
Population-weighted driving distance to 7 nearest on-premise alcohol outlets, by counties, United States, 2013

**Fig. 3:**
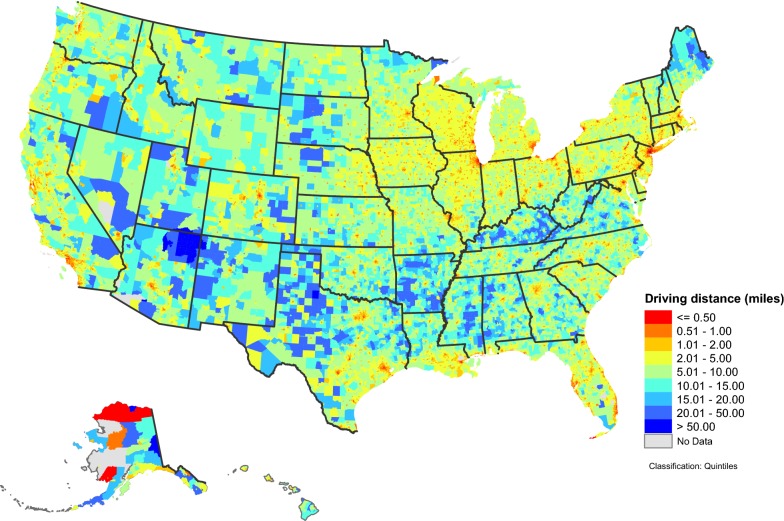
Population-weighted driving distance to 7 nearest on-premise alcohol outlets, by census tracts, United States, 2013

At the state-level (Table 3), population-weighted driving time ranged from 1.67 (DC) to 15.29 min (AR), with a median of 6.67 min (LA); population-weighted driving distance ranged from 0.69 (DC) to 7.91 (AR) miles, with a median of 2.95 miles (LA). In general, states in the northeast, the upper Midwest, and the western U.S. had a driving distance of less than 2 miles to the nearest 7 on-premise alcohol outlets (Fig. 1). The same pattern was observed for driving time (not shown). At the county level, population-weighted driving distance (Fig. 2) and population-weighted driving time (not shown) varied greatly by geography. Shorter population-weighted distances were observed in counties along the northeast coast, around the Great Lakes, in Florida, portions of the Rocky Mountain states, along the west coast, and in large metropolitan areas. Figure 3 presents detailed population-weighted driving distances at the census tract level. All data for 6 measures at state-, county- and census tract-level are included in Additional file 1.

**Table 3:** State-level population-weighted spatial accessibility measures to 7 nearest on-premise alcohol outlets, United States, 2013

State name	Census population 2010	Population-weighted accessibility			Population-weighted distance		
		Driving time	Driving distance	Euclidian distance	Driving time (min)	Driving distance (miles)	Euclidian distance (miles)
Alabama	4,779,736	1.34	3.25	4.93	11.92	5.77	4.02
Alaska	710,231	1.99	5.18	7.54	12.45	5.07	14.19
Arizona	6,392,017	2.46	6.22	9.68	7.33	3.44	2.45
Arkansas	2,915,918	1.20	2.82	4.16	15.29	7.91	5.78
California	37,253,956	3.37	8.51	13.17	3.79	1.56	1.01
Colorado	5,029,196	3.14	7.92	12.18	4.94	2.04	1.37
Connecticut	3,574,097	2.95	6.98	10.59	4.20	1.84	1.22
Delaware	897,934	2.56	6.07	8.93	5.19	2.40	1.66
District of Columbia	601,723	6.92	17.70	25.06	1.67	0.69	0.49
Florida	18,801,310	2.87	7.00	10.75	4.73	1.97	1.26
Georgia	9,687,653	1.66	4.00	6.44	8.71	3.92	2.65
Hawaii	1,360,301	3.19	8.05	11.96	6.73	2.98	2.16
Idaho	1,567,582	2.22	5.72	8.23	7.02	3.02	2.12
Illinois	12,830,632	4.26	10.89	16.61	3.49	1.48	1.02
Indiana	6,483,802	2.44	6.03	8.80	5.96	2.61	1.84
Iowa	3,046,355	3.08	8.07	11.34	5.32	2.25	1.62
Kansas	2,853,118	2.52	6.23	8.88	6.30	2.93	2.14
Kentucky	4,339,367	1.70	4.10	6.39	13.12	6.79	4.55
Louisiana	4,533,372	2.72	6.91	10.72	6.67	2.95	2.01
Maine	1,328,361	1.68	3.92	5.80	10.86	5.25	3.73
Maryland	5,773,552	2.92	7.33	10.78	4.79	2.02	1.31
Massachusetts	6,547,629	3.57	8.73	13.79	3.64	1.57	1.05
Michigan	9,883,640	2.66	6.59	9.41	5.38	2.25	1.59
Minnesota	5,303,925	2.58	6.32	8.92	6.09	2.60	1.85
Mississippi	2,967,297	1.20	2.96	4.37	14.71	7.36	5.44
Missouri	5,988,927	2.50	6.36	9.25	7.10	3.07	2.11
Montana	989,415	2.67	6.80	9.47	8.81	3.99	2.93
Nebraska	1,826,341	3.14	8.10	11.50	5.15	2.22	1.63
Nevada	2,700,551	3.03	7.76	12.96	4.08	1.66	1.11
New Hampshire	1,316,470	1.79	4.21	6.36	8.57	3.88	2.66
New Jersey	8,791,894	4.54	11.49	16.31	3.09	1.32	0.88
New Mexico	2,059,179	1.79	4.30	6.48	10.04	4.91	3.66
New York	19,378,102	5.68	14.94	25.19	2.94	1.21	0.80
North Carolina	9,535,483	1.56	3.83	5.94	9.16	4.11	2.77
North Dakota	672,591	2.67	6.86	9.81	7.92	3.47	2.58
Ohio	11,536,504	3.04	7.55	10.91	5.11	2.23	1.56
Oklahoma	3,751,351	1.89	4.80	6.99	8.61	4.09	2.88
Oregon	3,831,074	3.48	8.75	12.83	4.77	2.05	1.39
Pennsylvania	12,702,379	4.06	10.56	14.86	4.52	1.95	1.32
Rhode Island	1,052,567	4.52	10.82	16.08	2.89	1.29	0.86
South Carolina	4,625,364	1.68	4.11	6.36	7.94	3.48	2.36
South Dakota	814,180	2.44	6.42	8.83	9.11	4.07	3.19
Tennessee	6,346,105	1.56	3.84	5.86	9.78	4.34	2.89
Texas	25,145,561	2.56	6.45	10.00	6.06	2.76	1.91
Utah	2,763,885	1.94	4.66	6.95	6.60	3.03	2.26
Vermont	625,741	1.98	4.62	6.76	9.64	4.49	3.03
Virginia	8,001,024	1.94	4.65	7.07	8.29	4.01	2.64
Washington	6,724,540	2.98	7.61	11.02	5.30	2.20	1.45
West Virginia	1,852,994	2.02	5.01	7.47	10.69	4.97	3.05
Wisconsin	5,686,986	4.19	10.61	14.74	4.07	1.65	1.15
Wyoming	563,626	2.57	6.50	9.00	7.81	3.56	2.70

The mean estimates of population-weighted driving time, driving distance, and Euclidian distance all increased from large central metro to noncore categories (Table 4). Populations living in medium metro counties had shorter median driving time/distance and Euclidian distance than those living in large fringe metro counties. For the minimum of county-level population-weighted driving time, driving distance, and Euclidian distance, populations living in small metro counties had shorter driving time/distance and Euclidian distance than those living in medium metro counties. Noncore counties had shorter minimum distances than micropolitan counties, for both driving distance and Euclidian distance, but noncore counties had longer minimum driving time than micropolitan counties. For the maximum of county-level population-weighted driving time, driving distance and Euclidian distance, driving time increased with increased rurality; for driving distance, small metro and micropolitan counties had shorter distances than medium metro counties; small metro counties had shorter Euclidian distances than medium metro counties.

**Table 4:** On-premise alcohol outlets spatial accessibility measures by National Center for Health Statistics (NCHS) urban–rural classification scheme for counties, United States, 2013

Measures	NCHS urban/rural class name	Number of counties	Mean	Standard deviation	Minimum	Median	Maximum
*Population*-*weighted accessibility*							
Driving time	Large central metro	68	4.69	2.38	1.94	4.03	13.31
	Large fringe metro	368	1.79	1.03	0.23	1.58	6.92
	Medium metro	373	1.91	1.07	0.15	1.86	5.34
	Small metro	358	1.91	1.05	0.02	1.97	5.12
	Micropolitan	641	1.84	0.92	0.02	1.76	5.59
	Noncore	1333	1.27	0.96	0.00	1.01	8.71
Driving distance	Large central metro	68	12.02	6.55	4.81	10.06	36.67
	Large fringe metro	368	4.39	2.63	0.41	3.94	18.04
	Medium metro	373	4.72	2.82	0.22	4.70	14.20
	Small metro	358	4.75	2.75	0.11	4.90	12.66
	Micropolitan	641	4.52	2.45	0.04	4.26	15.07
	Noncore	1333	3.11	2.53	0.00	2.37	19.85
Euclidian distance	Large central metro	68	18.40	10.71	7.55	15.11	69.78
	Large fringe metro	368	6.57	3.96	0.56	5.83	25.44
	Medium metro	373	6.91	4.09	0.28	7.02	19.98
	Small metro	358	6.82	3.94	0.35	6.93	17.88
	Micropolitan	641	6.29	3.33	0.26	5.98	20.33
	Noncore	1335	4.18	3.34	0.00	3.20	24.43
*Population*-*weighted distance*							
Driving time (min)	Large central metro	68	2.79	1.22	0.71	2.75	5.86
	Large fringe metro	368	8.94	5.57	1.55	7.51	32.85
	Medium metro	373	9.55	6.68	2.31	7.04	44.26
	Small metro	358	10.40	7.49	1.85	7.86	48.03
	Micropolitan	641	10.88	6.93	2.82	9.09	63.44
	Noncore	1333	17.78	11.92	2.94	14.90	216.83
Driving distance (miles)	Large central metro	68	1.13	0.50	0.26	1.10	2.49
	Large fringe metro	368	4.02	2.84	0.69	3.22	17.55
	Medium metro	373	4.56	4.13	0.91	3.09	32.69
	Small metro	358	4.92	4.06	0.76	3.43	25.61
	Micropolitan	641	5.23	3.90	1.13	4.10	29.61
	Noncore	1333	8.97	6.78	0.99	7.07	92.26
Euclidian distance (miles)	Large central metro	68	0.73	0.32	0.13	0.68	1.55
	Large fringe metro	368	2.71	1.99	0.46	2.18	12.84
	Medium metro	373	3.19	3.23	0.61	2.14	26.85
	Small metro	358	3.52	3.18	0.51	2.30	20.49
	Micropolitan	641	3.80	3.04	0.80	2.95	26.86
	Noncore	1335	7.61	13.75	0.75	5.34	398.21

Population-weighted distance measures based on driving time, driving distance, or Euclidian distance were strongly correlated with each other (p < 0.01) (Table 5). Additionally, population-weighted accessibility measures were also strongly correlated with each other (p < 0.01). However, population-weighted distance measures and accessibility measures were moderately correlated to each other (p < 0.01).

**Table 5:** Pearson correlation coefficients for the 6 spatial accessibility measures to 7 nearest on-premise alcohol outlets, United States, 2013

Metric	Population-weighted accessibility			Population-weighted distance		
	Driving time	Driving distance	Euclidian distance	Driving time	Driving distance	Euclidian distance
*Population*-*weighted accessibility*						
Driving time	1.00	0.97	0.93	− 0.45	− 0.42	− 0.40
Driving distance		1.00	0.95	− 0.41	− 0.39	− 0.38
Euclidian distance			1.00	− 0.43	− 0.41	− 0.34
*Population*-*weighted distance*						
Driving time				1.00	0.97	0.93
Driving distance					1.00	0.97
Euclidian distance						1.00

All correlations were significant at p < 0.01

Table 6 presents the odds ratios (ORs) of excessive drinking for an interquartile range increase in population-weighted accessibility measures and population-weighted distance measures for on-premise alcohol outlets. The interquartile ORs are significantly larger than one, which means that an increased alcohol outlet access were associated with significantly increased risks for excessive drinking in the United States. In other words, the odds ratios represent the minimum risk increase when a person moves from one county with a lower access (first quartile) to alcohol outlets to another county with a higher access (fourth quartile) to alcohol outlets. Population weighted accessibility measures detected stronger associations (larger OR) with excessive drinking outcomes and were more sensitive to alcohol outcomes than population weighted distance measures. Among these spatial accessibility measures, driving distance/time and Euclidian distance had similar sensitivities to binge drinking/heavy drinking. Among the population-weighted distance measures, driving distance/time-based ones were much more sensitive to binge drinking/heavy drinking than one based on Euclidian distance.

**Table 6:** The odds ratios (ORs) of excessive drinking for an interquartile range increase in population-weighted access measures to nearest 7 on-premise alcohol outlets, United States, 2013

Measures	Binge drinking OR^a^ (95% CI)	Heavy drinking OR^a^ (95% CI)
*Population*-*weighted accessibility*		
Driving distance	1.13 (1.11,1.15)	1.16 (1.13,1.19)
Driving time	1.13 (1.11,1.15)	1.16 (1.13,1.19)
Euclidian distance	1.12 (1.10,1.14)	1.15 (1.12,1.17)
*Population*-*weighted distance*		
Driving distance	1.11 (1.09,1.14)	1.15 (1.12,1.19)
Driving time	1.10 (1.08,1.13)	1.14 (1.11,1.18)
Euclidian distance	1.01 (1.00,1.02)	1.02 (1.00,1.03)

^a^The ORs were based on an interquartile range scale (the absolute difference between first and third quartiles), which means the odds increase for risky drinking behaviors when the county-level on-premise alcohol outlet access increases from first quartile to third quartile

## Discussion

Our study has demonstrated a robust approach to quantify spatial accessibility of geographic entities from a population health perspective. The population-weighted bottom-up approach provides great flexibility in generating spatial accessibility measures at any geographic level that could be linked with population health outcomes of interest. This is the first nationwide network-based analysis of spatial accessibility to on-premise alcohol outlets at the level of the census block and their aggregated measures provide a more holistic and accurate picture of population spatial accessibility to on-premise alcohol outlets from the local to the national level.

Census block-based population-weighted distance measures are very flexible and can be aggregated to any geographic level as needed (e.g., ZIP Code, neighborhood). The method accounts for uneven local population distributions, reduces the ecological bias in measuring alcohol outlet density, and is flexible in that it can incorporate more information when detailed data are available (e.g. size of the outlets, sales of the outlets). Population-weighted accessibility measures can be linked with other health outcomes at different geographic levels to study the relationship of alcohol access to related harms. Additional analysis is needed to evaluate its sensitivity to excessive drinking outcomes and related population health outcomes. Additional analysis is also needed to evaluate the difference between commercial datasets of licenses alcohol outlets with local alcohol license data.

In spatial interaction modeling and spatial choice modeling, how to characterize destination or spatial choice set is still challenging and has no certain answer. We used 7 on-premise alcohol outlets in our model based on Miller et al. and Saaty et al. [13, 14]. We give different probabilities for those 7 outlets based on the Huff model [4]. In this approach, the population in any given census block has a higher probability of accessing a nearer rather than farther on-premise alcohol outlet, and it also accounts for people who may not always chose the nearest outlet.

This study has several limitations. First, alcohol outlet size was treated as one unit (e.g., all alcohol outlets are equally weighted). Second, commercial datasets may not be updated frequently enough to reflect local businesses opening and closing, which may result in over- or under-estimations of spatial accessibility to on-premise alcohol outlets in some locations. Third, we were aware that people often purchase alcohol from off-premise alcohol outlets (stores) and consume the alcohol at home. Due to lack of data access, we could not include off-premise alcohol outlet in our analysis. This analysis is not meant to present the entire picture of access for both on- and off-premise alcohol outlets. Further analysis may be conducted to include off-premise alcohol outlets in the model to obtain a more comprehensive measurement of the impact of alcohol outlet access, once such data becomes available. Finally, census block population counts are only available every 10 years when the decennial census is conducted. For the non-decennial years, census block population could be updated via small area population estimation techniques.

Population-weighted spatial accessibility index and population-weighted distance metrics are moderately correlated, which indicates that the population-weighed spatial accessibility index is conceptually different from population-weighted distance metrics and captures different aspects or dimensions of spatial accessibility to target destinations. Epidemiologically, we can expect that the population-weighted accessibility measures and population-weighted distance measures could have differential sensitivity to population health outcomes. Our sensitivity analysis between access to on-premise alcohol outlets and excessive drinking suggested that less-intuitive spatial accessibility measures are much more sensitive than those more-intuitive direct distance measures. It suggests that some conclusions in the current literature on alcohol outlet density and adverse health outcomes could underestimate the magnitude of associations between alcohol outlet density and harmful drinking behaviors. These more-sensitive alcohol outlet density measures could be used in public health impact studies on alcohol outlets, provided data with the necessary spatial granularity are available. Alcohol use is a complicated human behavior and access to on-premise alcohol outlets is only one of many potential explanatory factors. It is also challenging to quantify people’s preference and selection of on-premise alcohol outlets. Our study method accounts for the probability that closer outlets get more access than more distant outlets, but also takes into consideration that people may not always go to the closest outlets as indicated by a recent USDA report (EIB-138) [15].

## Conclusions

These results can be used to assess the relationship between geographic access to on-premise alcohol outlets and health outcomes. Additionally, this study demonstrated a flexible and robust method that can be applied or modified to quantify spatial accessibility to public resources, such as stores that serve healthy food options, medical care providers, and parks and greenspaces, and spatial exposure to local adverse environments such as tobacco stores and fast food restaurants. This spatial accessibility modeling framework could be conveniently applied to any countries or places with hierarchical census geography. In modern society, census geographic hierarchy is the base for socioeconomic and population statistics. Our bottom-up approach within a census geographic hierarchy could be widely applicable and substantially improve the accuracy and flexibility in quantifying population spatial access to public health resources or exposure to environmental hazards of interest.

## Additional file

**Additional file 1:** Population-weighted spatial access to 7 nearest on-premise alcohol outlets by U.S. states, counties and census tracts, 2013.

## Abbreviations

CDC: The Centers for Disease Control and Prevention
PWA: population-weighted accessibility
PWD: population-weighted distance
HSIP: The Homeland Security Infrastructure Program
NAICS: The North American Industry Classification System
NCHS: The National Center for Health Statistics
MSA: metropolitan statistical area
